# Genetic variants affecting human TRPA1 or TRPM8 structure can be classified *in vitro* as ‘well expressed’, ‘poorly expressed’ or ‘salvageable’

**DOI:** 10.1042/BSR20150108

**Published:** 2015-10-08

**Authors:** Kevin Morgan, Laura Rachel Sadofsky, Alyn Hugh Morice

**Affiliations:** * Centre for Cardiovascular and Metabolic Research, Hull-York Medical School, University of Hull, Daisy Building, Castle Hill Hospital, Cottingham, Hull HU16 5JQ, U.K.

**Keywords:** expression levels, human TRP channels, single nucleotide polymorphisms (SNPs)

## Abstract

Multiple mis-sense variants of TRPA1 (transient receptor potential A1) and TRPM8 (transient receptor potential M8) are recorded in the human genome single nt polymorphism (SNP) database, but their potential impact on channel signalling in patho-physiology is not fully explored. Variants, mostly quite rare in the general human population, alter sites in different structural domains of these homo-tetrameric ion channel proteins. The effects of individual SNPs affecting the large cytoplasmic N-terminal domain have not been completely documented for TRPM8 or TRPA1. We examined the Ca^2+^ signalling properties of a short-list of eight variants affecting the N-terminal domain by individual expression in human embryonic kidney HEK293 or neuroblastoma (SH-SY5Y) cell lines (four SNP variants for TRPM8: G150R, K423N, R475C, R485W and four for TRPA1: Y69C, A366D, E477K, D573A). These were compared with TRPA1 SNP variants affecting intracellular loops located beyond the N-terminal domain and associated with gain of function (such as increased sensitivity to agonists: TRPA1 R797T and N855S). A substitution in TRPA1 (Y69C) exhibited high expression/sensitivity to agonists (high iCa^2+^max (maximum level of intracellular calcium ion), similar to R797T, but less sensitive than N855S), whereas each of the other non-conservative substitutions exhibited poor signalling response (low iCa^2+^max). Responses from these poorly expressed variants could be salvaged, to different extents, by pre-treating cells with the Src (Src protein) family inhibitor protein kinase inhibitor PP2 (PP2: 4-Amino-3-(4-chlorophenyl)-1-(t-butyl)-1H-pyrazolo[3,4-d]pyrimidine, 4-Amino-5-(4-chlorophenyl)-7-(t-butyl)pyrazolo[3,4-d]pyrimidine), or with micromolar Zn^2+^. The TRPA1 variants and several experimental mutants (TRPA1 Y97F, Y226F and YY654–655FF) expressed poorly in SH-SY5Y compared with HEK293 cells. More in-depth studies are needed to identify SNP variants eliciting gain of function in these TRP (transient receptor potential) channels and to assess their roles in medical conditions.

## Abstract (precis)

Genetic variants of human transient receptor potential channels A1 and M8 expressed in human embryonic kidney HEK293 and SH-SY5Y cells were assayed using Ca^2+^ signalling. TRPA1 Y69C responded well. Poorly expressed variant signalling was enhanced by pre-treatment with tyrosine kinase inhibitor PP2 or Zn^2+^.

## INTRODUCTION

The cold-temperature-sensitive TRPA1 (transient receptor potential A1) and TRPM8 (transient receptor potential M8) Na^+^, Ca^2+^ ion channels are interesting pharmacological targets due to their roles in sensory nerve activation, pain and inflammation [1,2]. These channels are formed as homo-tetrameric complexes, with each subunit containing approximately 1100 amino acid residues. Data from the human genome sequencing project indicates the existence of multiple genetic variants altering the code for single amino acid residues distributed across the length of the TRPA1 and TRPM8 genes. Approximately 130 single residues TRPA1 and 200 TRPM8 variants are currently recorded, including allele frequency data for 30%–40% of these [see summaries in NCBI single nt polymorphism (SNP) database]. A ‘gain-of-function’ variant affecting TRPA1 (N855S) associated with episodic pain syndrome was identified recently [3–5]. Genetic-association studies are emerging for TRPM8 in relation to airway physiology [6,7]. Previous discoveries regarding structure–function relationships in TRPA1 E179K, TRPM8 and heat-sensitive TRPV1 (transient receptor potential V1), and the information regarding gene sequence variation, suggest that further screening of the properties of variants may aid implementation of targeted clinical studies [3–9].

Hypothetically, some variants may be expected to possess differences in various aspects of their poly-modal regulation and exogenous agents, such as zinc ions (Zn^2+^), may modify their behaviour [10], perhaps with relevance to particular physiological circumstances [11]. However, the functional properties of most of the variants have not been reported. We recently described increased sensitivity to agonists for TRPA1 R797T and interesting modulatory effects of PP2 on cells transfected with TRPA1 or TRPM8 [12]. Therefore, we initiated a preliminary survey of the properties of variants that alter single residues in the large N-terminal domains of each channel and characterized them in terms of agonist-induced transient effects on intracellular Ca^2+^ levels.

Prioritization of which of the multiple variants listed in the SNP database to study was informed by protein structural information, but remained relatively simplistic. For TRPM8, we selected variants representing four non-conservative substitutions located in two evolutionarily conserved sections of the N-terminal domain. Structure–function studies for TRPM8 have focused mainly on amino acid residues in the transmembrane bundle [5,13–19] rather than the N-terminal domain [20], although a potential structure for the latter has been proposed as part of a whole molecule model [21].

For TRPA1, we selected variants representing non-conservative substitutions located in the ankyrin repeat (AR) sequences (Y69C in AR1, A366D in AR9, E477K in AR12 and D573A in AR16). The ARs form short helices assembled into an asymmetrical cylindrical tertiary structure possessing a surface available for protein interactions [22]. The influence of alterations within the individual ARs is relatively unexplored [23,24] compared with studies examining the transmembrane bundle [25–29] or C-terminal domain [30,31].

By electing to study SNPs causing non-conservative substitutions, we expected to observe evidence for altered channel properties.

## EXPERIMENTAL

### Reagents and chemicals

Most reagents were purchased from Sigma–Aldrich or Tocris, including WS 12 (4-methoxyphenyl-5-methyl-2-1-methylethyl-cyclohexanecaboxamide), menthol, AMTB (*N*-3-aminopro-pyl-2-3-methylphenyl-methoxy-*N*-2-thienylmethylbenzamide) hydrochloride, allyl-isothiocyanate (AITC), cinnamaldehyde, carvacrol, eugenol and A967079 (4-fluorophenyl-2-methyl-1-pentene-3-one oxime). Stock solutions of agonists and antagonists were prepared in DMSO and were stored at–20°C (except for A967079, stored at 4°C).

### Bioinformatics

The NCBI database and the 1000 genomes deep catalogue of human genetic variation (http://browser.1000genomes.org/index.html) were used to access human gene and cDNA sequences and SNP data for TRPA1 and TRPM8 (http://www.ncbi.nlm.nih.gov/snp/). The NetPhos2.0 (www.cbs.dtu.dk/services/NetPhos/) and Scansite (scansite.mit.edu/) servers were used to locate putative tyrosine phosphorylation sites in each TRP (transient receptor potential) channel. Clustalw2 (http://www.ebi.ac.uk/Tools/msa/clustalw2/) was used to align the primary sequence of TRPM8 with TRPM2.

### DNA manipulation

A series of mutations were made at sites affecting the structure of human TRPM8 or TRPA1 (Figures 1a and 3a). Oligonucleotides were purchased from Eurofins-MWG-Operon. Mutated TRP channel cDNAs were created in plasmid pcDNA3.1neo(+) using quick-change PCR mutagenesis with velocity DNA polymerase (Bioline)-mediated amplification. Methylated template DNA was removed by digestion with DpnI (Promega) and mutated plasmids were cloned and isolated following transformation of competent *Escherichia coli* (Bioline). DNA sequences were confirmed using automated sequencing (Eurofins Genomics) with appropriate primers.

**Figure 1:**
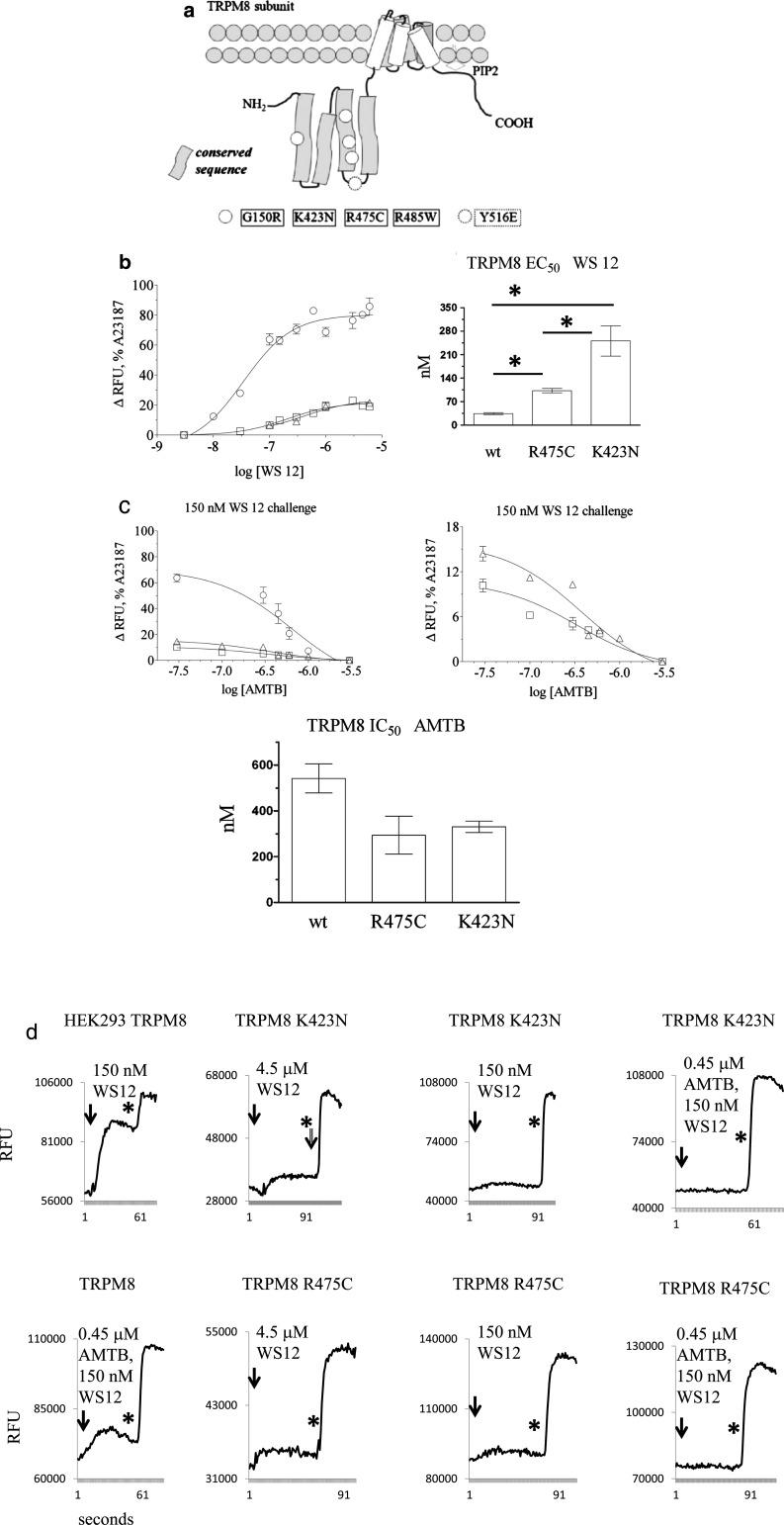
Properties of TRPM8 variants in HEK293 cells (**a**) Line drawing representing the subunit structure of TRPM8, with the approximate locations of genetic variants (G150R, K423N, R475C, R485W) and the experimental mutant (Y516E) examined in the present study. Shaded ribbons in the N-terminal domain represent evolutionarily conserved regions identified using sequence alignment with the closest relative TRPM2. (**b**) Examples of dose-response curves generated from quantitative analyses of changes in intracellular Fluo3- Ca^2+^ fluorescence elicited by exposure of transfected HEK293 cells to TRPM8 agonist WS 12, standardized to the maximum fluorescence elicited following addition of 2 μM ionophore A23187. Estimates of EC_50_ values were made by analysing measurements generated from three separate experiments with each data-point treatment performed in triplicate. The WS 12 EC_50_ value ± S.D. for non-mutated TRPM8 (circle data points) was 34 ± 5 nM, for R475C 103 ± 12 nM (square data points) and for K423N 251 ± 78 nM (triangle data points). The histogram indicates that differences between variants were statistically significant (*P < 0.05). (**c**) Examples of the dose-dependent inhibition of responses to 150 nM TRPM8 agonist WS 12 by pre-exposure of transfected HEK293 cells to the antagonist AMTB. Data for estimates of IC_50_ from treatments performed in triplicate on three separate occasions and include ± S.D. The AMTB IC_50_ value for non-mutated TRPM8 was 0.5 ± 0.1 μM, for R475C 0.3 ± 0.1 μM and for K423N 0.4 ± 0.1 mM (symbols as 1b). Apparent differences did not reach statistical significance. Right-hand graph illustrates curves when *y*-axis is expanded for analysis of relatively weak responses. (**d**) Examples of real-time changes in intracellular Fluo3-Ca^2+^ fluorescence [RFU, plotted versus time (seconds)] in transfected HEK293 cells treated with TRPM8 agonist WS 12 and antagonist AMTB. Addition of ionophore A23187 indicated by a down-arrow with asterisk (*).

### Cell culture, transfection and clone isolation

Human embryonic kidney (HEK)293 cells were cultured on matrigel-coated plasticware. HEK293 or SH-SY5Y cells grown in Dulbecco's modified Eagle's medium (DMEM), 10% fetal calf serum supplemented with glutamine were transfected in 6 cm diameter culture dishes using DNA mixed with Fugene 6 (Promega) in Optimem I (Invitrogen) and multiple distinct clones for each construct were isolated using glass cloning cylinders (Sigma–Aldrich) following selection with G418. On average, eight clones for each construct were screened for response to TRP channel agonists.

### Intracellular Ca^2+^ assay

Cells detached from culture flasks using HEPES buffered saline, pH 7.4, containing 1.7 mM EDTA were diluted with 10 volumes PBS, pelleted by centrifugation and loaded with Fluo-3AM (2.2 μg/ml) at room temperature for 25 min in HEPES-buffered assay solution, pH 7.4, (containing physiological NaCl, KCl, MgCl_2,_ glucose and 1 mM CaCl_2_ without BSA, sulfinpyrazone or probenecid). The cells were washed with PBS and re-suspended in HEPES-buffered assay solution, pH 7.4, for fluorescence measurements performed, as described previously [12].

### Quantitative and statistical analyses

Measurements of peak amplitudes from real-time traces of Fluo3-Ca^2+^ fluorescence were collected and analysed to determine mean change in relative fluorescence units (ΔRFU) with S.D. compared with the effect of 2 μM ionophore (A23187). Statistical significance of differences between clones or responses to different agents was calculated using Student's *t*test or ANOVA with Tukey's post-hoc honest significant difference test (using Graphpad Prism software or online tools). Statistically significant differences were denoted by an asterisk in graphical summaries of data (*P < .05 in all cases and *P* < 0.01 in most cases).

## RESULTS

### Several TRPM8 variants appeared to be poorly expressed in transfected HEK293 cells compared with non-mutated TRPM8

Mutated and sequence-validated cDNA expression constructs generating the TRPM8 variants G150R, K423N, R475C, R485W and the experimental mutant Y516E were individually transfected into HEK293 cells. Figure 1(a) indicates the location of mutated SNP residues in regions of evolutionarily conserved sequence in the protein subunit and Y516E in a putative connecting region. Multiple G418 resistant clones were isolated for each variant and characterized alongside a clone expressing non-mutated TRPM8 (‘wild-type’ or ‘reference sequence’). The responses of clones to different concentrations of specific agonist (WS 12 or menthol), including pre-treatments with antagonist (AMTB), were recorded in measurements of intracellular Fluo3-Ca^2+^ and were compared with each other and to responses from untransfected cells (Figures 1b and 1c; Supplementary Data). The results indicated significant differences in maximum achievable amplitude of fluo3-Ca^2+^ signal (iCa^2+^max) in response to WS 12 for clones expressing the different constructs. For multiple clones isolated and screened, each of the SNP variants exhibited weaker responses to agonist compared with cells expressing non-mutated wild-type TRPM8. (Un-transfected HEK293 cells did not exhibit intracellular Ca^2+^ signals in response to WS 12 or menthol; see Supplementary Data).

Although iCa^2+^max (maximum level of intracellular calcium ion) values were relatively low, it was possible to accurately estimate agonist EC_50_ values and antagonist IC_50_ values for variants K423N and R475C (Figures 1b and 1c). The right-side dose-response curve in Figure 1(c) demonstrates an expanded *y*-axis for the data in the left-side graph. The apparent EC_50_ for TRPM8 R475C response to WS12 was elevated approximately 3-fold relative to non-mutated TRPM8 and that for K423N was elevated approximately 7-fold. Both TRPM8 R475C and K423N appeared to be marginally more sensitive to the antagonist AMTB compared with non-mutated TRPM8, with K423N apparently more sensitive to antagonist than R475C, although differences failed to reach statistical significance (see bar graphs in Figures 1b and 1c). Representative examples of real time fluo3-Ca^2+^ signals are presented in Figure 1(d).

Responses to TRPM8 agonists from cells transfected with the variants G150R or R485W or with the experimental mutant Y516E were undetectable. However, responses to agonist could be detected from these cells after a short pre-treatment with low micromolar concentrations of PP2 or divalent zinc (Zn^2+^, added from 1000× concentrated stock solutions of ZnCl_2_; Figure 2). These agents have previously been shown to modify signals from TRP channels in a mutation-specific fashion. Although relatively high doses of WS 12 were required to elicit responses from these variants after treatment with PP2 (Figure 2a) or Zn^2+^, the responses were agonist dose-dependent (Figure 2b).

**Figure 2:**
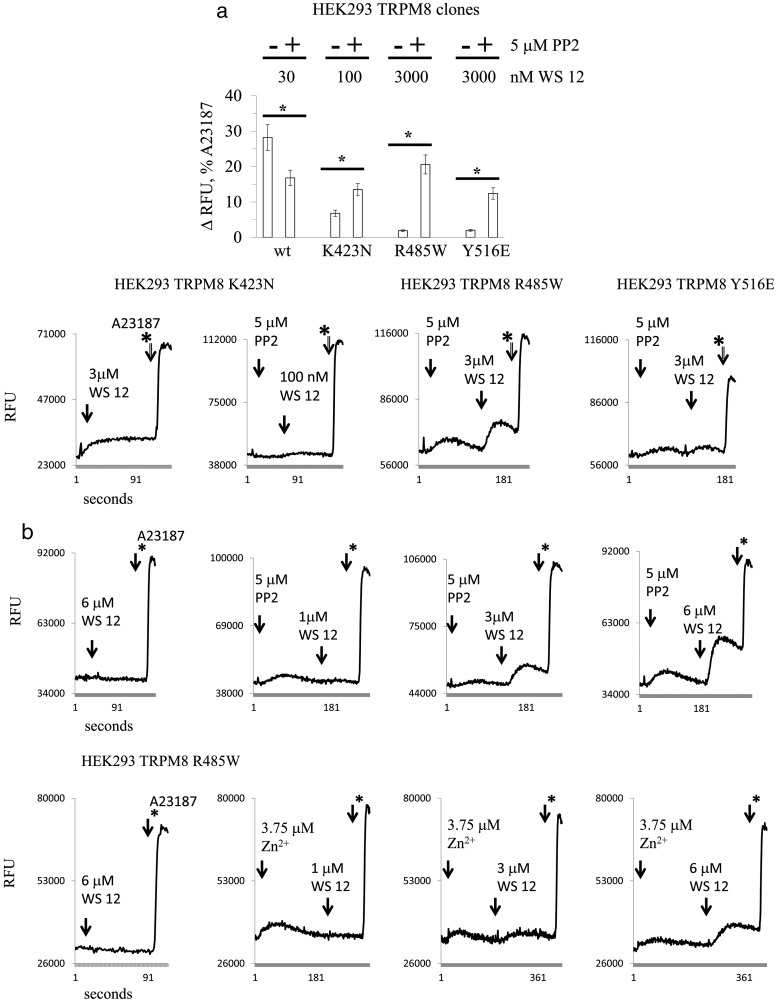
Effects of PP2 or Zn^2+^ on TRPM8 variant signalling (**a**) PP2 pre-treatment of transfected HEK293 cells elicited differential effects on responses of TRPM8 variants to agonist WS 12. The histogram summarizes results from experiments performed in triplicate on three separate occasions and include ± S.D. PP2 inhibited responses from non-mutated TRPM8 [wild-type (wt)] but enhanced responses from SNP variants or experimental mutantY516E, as indicted in representative fluorescence traces (below). (**b**). Pre-treatment with 3.75 μM Zn^2+^ also enhanced intracellular Ca^2+^ responses to agonist WS 12 from TRPM8 variants K423N, R475C, R485W and experimental mutant Y516E, as exemplified by fluorescence traces from relatively abundant allele R485W compared with responses from PP2-treated cells.

Brief pre-treatment of cells with 5 μM PP2 enabled detection of agonist-induced responses ranging from 4% to 22% of the ionophore (A23187)-induced maximum Fluo3-Ca^2+^ signal in a mutant-specific fashion. This effect is expressed relative to signal from wild-type TRPM8 in Table 1. Addition of Zn^2+^ to Fluo-3-loaded cells caused a dose-dependent transient increase in fluorescence during assays but it also quenched the maximum signal elicited following addition of A23187 (result not shown). These effects did not prevent qualitative detection of enhanced signals from these poorly responsive clones (Figure 2b), but the quenching effect precluded standardized quantitative assessment.

**Table 1:** Comparisons of sensitivity to agonist and degree of salvage by PP2 Properties of human TRPM8 and TRPA1 SNP variants and experimental mutants expressed in HEK293 cells, including relative effects ± S.D. where indicated (*n*=3, on three separate occasions).

	Sensitised	No change	Inactivated	Degree of salvage using PP2
ΔRFU Fluo3-Ca^2+^ % relative to amplitude from non-mutated TRP channel				
TRPM8	–	–	G150R	∼7% (at 6 μM WS 12)
	–	–	K423N	21 + 4% (at 100 nM WS 12)
	–	–	R475C	57 + 7% (at 3 μM WS 12)
	–	–	R485W	44 + 7% (at 6 μM WS 12)
Experimental				
mutant	–	–	Y615E	24 + 5% (at 6 μM WS 12)
TRPA1	Y69C	–	–	Not enhanced by PP2
	N855S	–	–	Not enhanced by PP2
	R797T	–	–	∼113% (at 3 μM AITC)
	–	–	A366D	12 + 5% (at 10 μM AITC)
	–	–	E477K	10 + 3% (at 10 μM AITC)
	–	–	D573A	∼2% (at 10 μM AITC)
Experimental				
mutant	–	Y97F		Elevated ∼10%–15% by PP2 (at 6 μM AITC)
				But no relative increase compared with non-mutated control
	–	Y226F	–	As Y97F
	–	YY654–655FF	–	As Y97F

Interestingly, pre-treatment of cells expressing TRPM8 K423N with PP2 or Zn^2+^ did not result in enhanced response to agonist (result not shown) and non-mutated TRPM8 responses were inhibited by pre-treatment with PP2 (Figure 2a).

The effects of TRPM8 SNP variants on iCa^2+^signalling can be compared and contrasted with those occurring with TRPA1 SNP variants.

### Certain TRPA1 variants and tyrosine-to-phenylalanine mutants exhibited good function compared with non-mutated TRPA1 in transfected HEK293 cells

Mutated cDNA expression constructs generating the TRPA1 variants Y69C, A366D, E477K and D573A were individually transfected into HEK293 cells. The locations of mutations in the TRPA1 subunit are depicted in Figure 3(a). Multiple G418 resistant clones were isolated for each variant and characterized alongside clones expressing non-mutated TRPA1 and the ‘gain-of-function’ variants N855S and R797T in addition to un-transfected cells (Figure 3; Supplementary Data).

**Figure 3:**
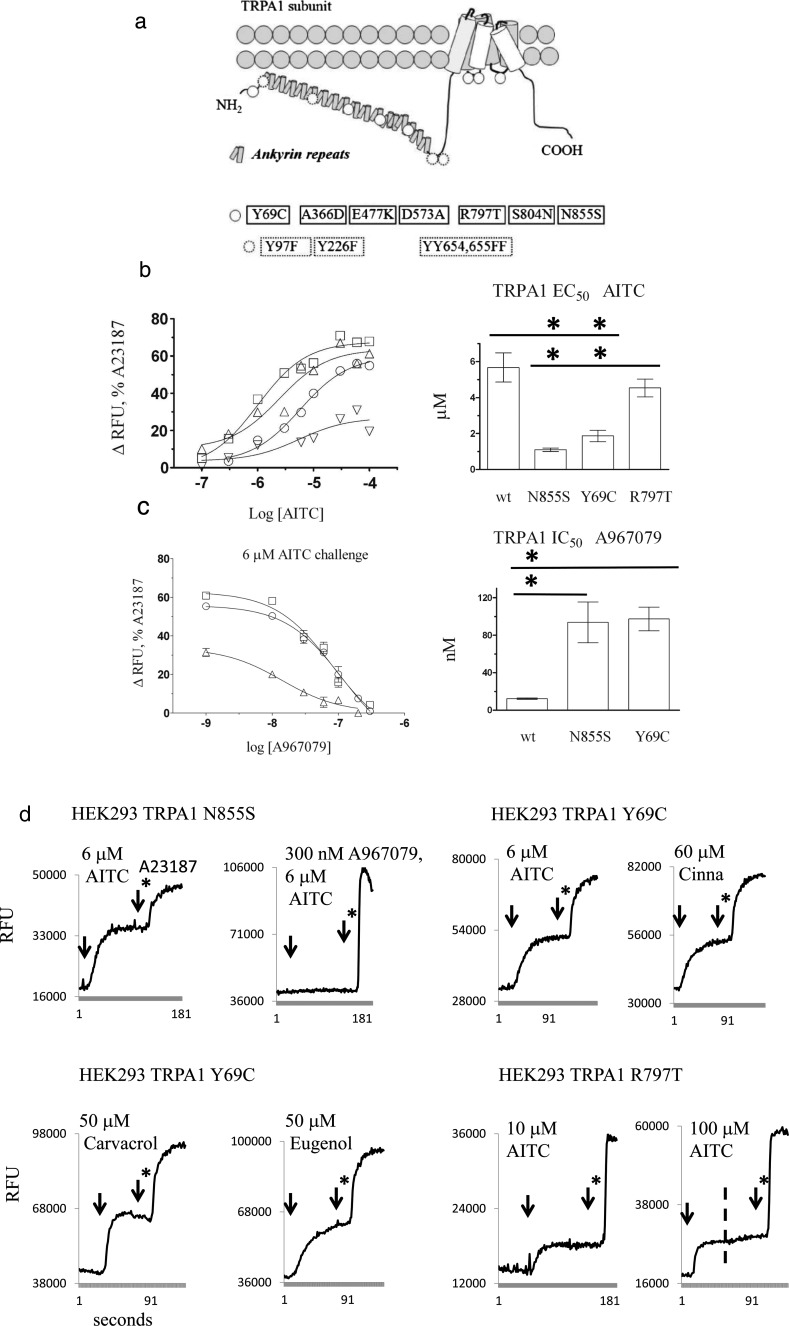
Properties of TRPA1 variants in HEK293 cells (**a**) Line drawing representing the subunit structure of TRPA1 and the approximate locations of genetic variants (Y69C, A366D, E477K, D573A, R797T, S804N, N855S) and the experimental mutants (Y97F, Y226F, YY654–655FF) examined in the present study. (**b**). Examples of dose-response curves generated from quantitative analyses of changes in intracellular Ca^2+^-Fluo3- fluorescence elicited by exposure of transfected HEK293 cells to TRPA1 agonist AITC, standardized to the maximum fluorescence elicited following addition of 2 μM ionophore A23187. Estimates of EC_50_ values were made by analysing measurements generated from three separate experiments with each data-point treatment performed in triplicate ± S.D. The AITC EC_50_ value for non-mutated TRPA1 was 5.7 ± 1.4 μM (circle data points), for N855S it was 0.9 ± 0.3 μM (square data points), for Y69C it was 1.9 ± 0.6 μM (open-triangle data points) and for R797T it was 4.5 ± 0.9 (black triangle data points). Histogram indicates statistically significant differences (*P < 0.05). (**c**) Examples of the dose-dependent inhibition of responses to 6 μM TRPA1 agonist AITC by pre-exposure of transfected HEK293 cells to the antagonist A967079. Data for estimates of IC_50_ from treatments performed in triplicate on three separate occasions ± S.D. The A967079 IC_50_ value for non-mutated TRPA1 was 12 ± 1 nM (triangles), for N855S it was 94 ± 38 nM (squares), for Y69C it was 97 ± 22 nM (circles). Histogram indicates statistically significant differences (*P < 0.05). (**d**) Examples of real-time changes in intracellular Fluo3-Ca^2+^ fluorescence in transfected HEK293 cells treated with TRPA1 agonists (AITC, cinnamaldehyde, carvacrol, eugenol) and antagonist A967079. Note: unstable peak (to right of dotted vertical line) elicited by high-dose AITC (100 μM), suggesting an off-target effect.

TRPA1 agonist concentrations (AITC, cinnamaldehyde, carvacrol and menthol) and antagonist (A967079) were carefully titrated to elicit signals for measurements of intracellular Ca^2+^ from multiple clones. Responses were compared between different clones expressing the same or different mutated channel.

HEK293 cells expressing the variants N855S, Y69C and R797T responded well to TRPA1 agonists (iCa^2+^max comparable to non-mutated TRPA1) and it was possible to estimate apparent agonist EC_50_ and antagonist IC_50_ values using measurements of peak amplitudes of intracellular Fluo3-Ca^2+^ (Figures 3b and 3c). Clones expressing these particular TRPA1 variants exhibited apparently increased sensitivity to agonists relative to non-mutated TRPA1, with reductions in estimated EC_50_ values of approximately 5-, 3- and 1.3-fold respectively. Differences in apparent sensitivities to the antagonist A967079 were also detected (Figures 3b and 3c), suggesting that N855S and Y69C may both be ∼8-fold less sensitive than non-mutated TRPA1. Agonist potency followed the order: AITC > cinnamaldehyde > carvacrol > menthol in all cases (Figure 3d).

The variants A366D and E477K exhibited poor responses to agonists (relatively low iCa^2+^max) that could be enhanced using brief pre-treatment of cells with PP2 (Figure 4), similar to the situation with TRPM8 SNP variants (Figure 2). Interestingly, the highly responsive TRPA1 variants N855S and Y69C were not affected by pre-treatment with PP2. Higher doses of agonist were required to elicit responses from TRPA1 A366D and E477K compared with TRPA1 N855S and Y69C. The apparent EC_50_ value for activation of TRPA1 A366D by AITC was estimated to be ∼76 μM, approximately 13-fold higher than for non-mutated TRPA1. Pre-treatment of cells expressing either variant with micromolar concentrations of PP2 or Zn^2+^ elicited modest, but detectable increases in signal following addition of agonist, reproducibly elevating the signal in average up to 10% (A366D) or 4% (E477K) of the ionophore (A23187)-induced maximum Fluo3-Ca^2+^ signal with moderate doses of AITC (10 or 20 μM). The effect of PP2 is presented relative to responses from wild-type TRPA1 and compared with TRPM8 variants in Table 1.

**Figure 4:**
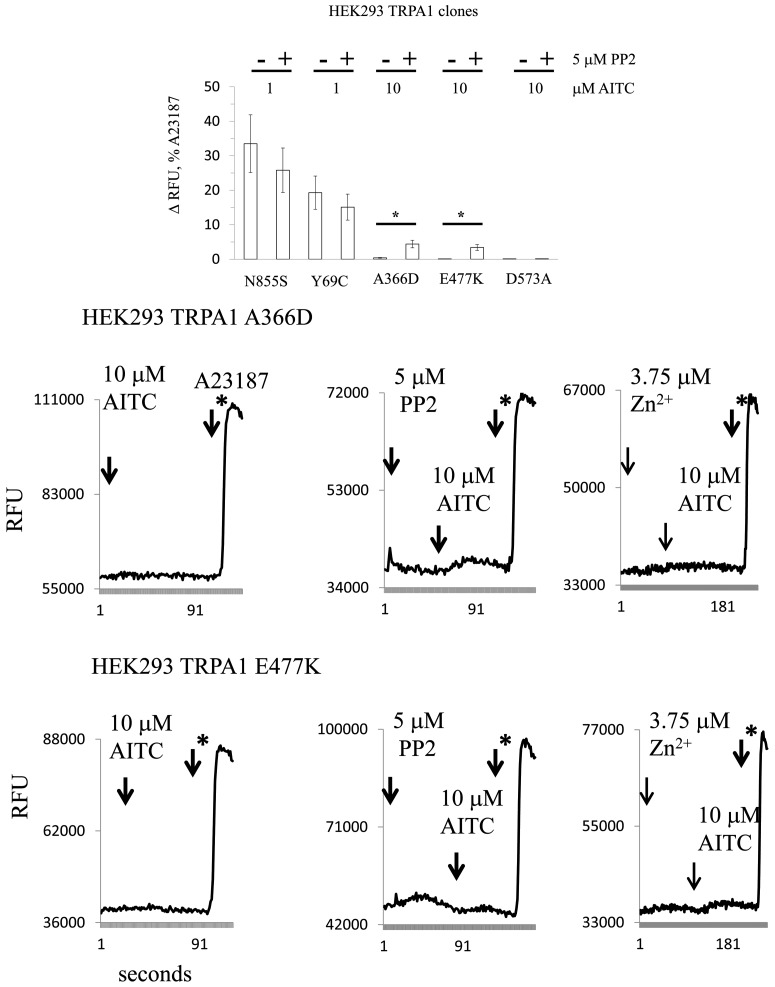
Effects of PP2 or Zn^2+^ on TRPA1 variant signalling Responses from TRPA1 variants that responded poorly to agonists (A366D, E477K) could be enhanced by pre-treatment of transfected HEK293 cells with PP2 or Zn^2+^. Experiments performed in triplicate on three separate occasions ± S.D., asterisks on histogram indicate statistically significant differences, *P* < 0.05.

Responses to agonist from cells transfected with TRPA1 D573A were very small (almost undetectable) and were hardly affected by PP2 or Zn^2+^. In contrast, responses to low-dose agonist (1 or 3 μM AITC) from TRPA1 N855S or Y69C were not enhanced by pre-treatment by PP2 or Zn^2+^, but responses from TRPA1 R797T to 3 μM AITC were modestly enhanced by 5 μM PP2.

Several experimental mutants in which tyrosine residues were converted into phenylalanine residues each responded well to agonist (Y97F, Y226F and YY654–655FF), with sensitivities similar to non-mutated TRPA1. Responses from these mutants could be enhanced using PP2 (Supplementary Figure S1b; Figure 5). (Untransfected HEK293 cells did not exhibit intracellular Ca^2+^ signals in response to TRPA1 agonists, with the exception of off-target effects of AITC when applied at 60 μM or higher; see Supplementary Data).

**Figure 5:**
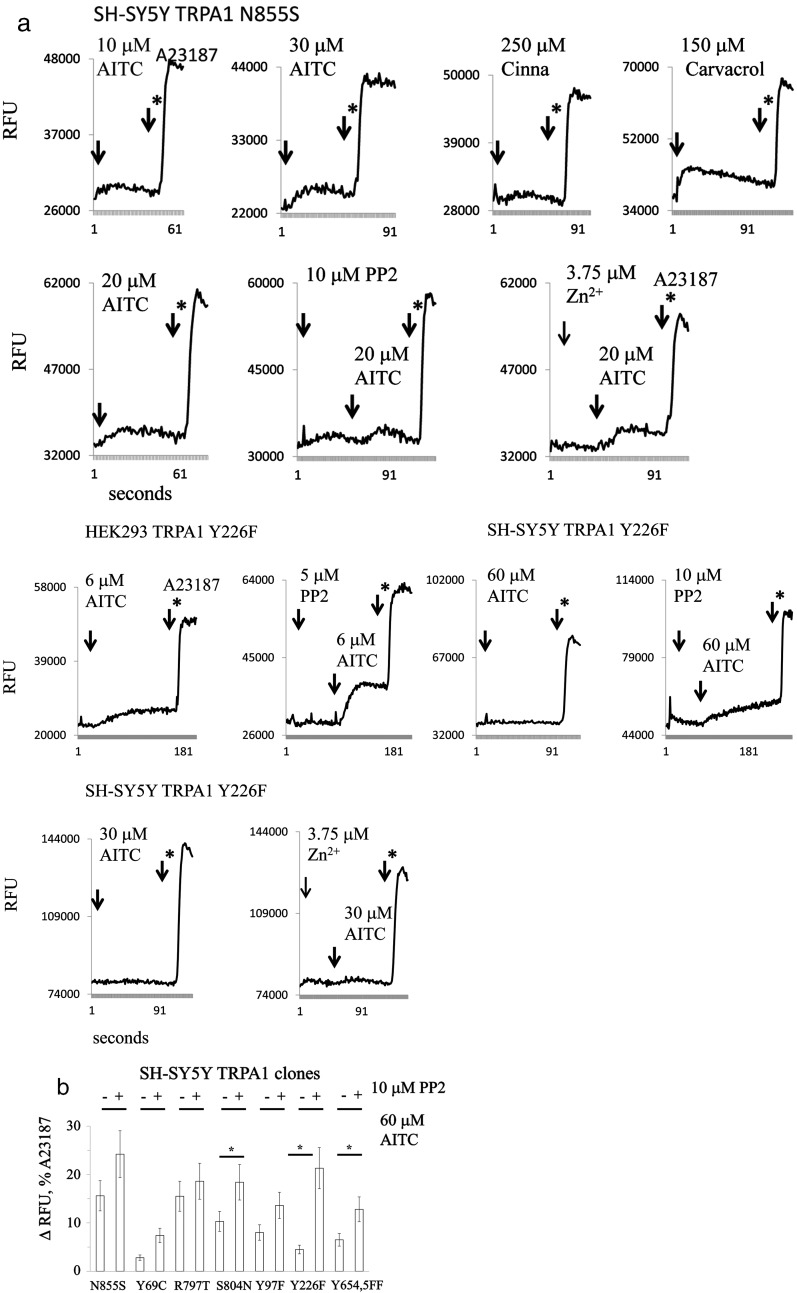
Properties of TRPA1 variants and experimental mutants compared in HEK293 and SH-SY5Y cells (**a**) SH-SY5Y cells transfected with TRPA1 variants or experimental mutants exhibited weaker responses than transfected HEK293 cells, as exemplified by TRPA1 N855S and Y226F. Responses from TRPA1 expressed in transfected SH-SY5Y cells could be enhanced by pre-treatment with PP2 or addition of 3.75 μM Zn^2+^. (**b**) Histogram represents data from experiments performed in triplicate on three separate occasions ± S.D. and asterisks denote statistically significant differences, *P* < 0.05.

### TRPA1 variants and tyrosine-phenylalanine mutants responded poorly in transfected SH-SY5Y cells

When SH-SY5Y cells were transfected with TRPA1 expression constructs, multiple G418-resistant clones responded only poorly to agonists compared with transfected HEK293 cells. Responses from TRPA1 N855S were small but detectable using moderate doses of AITC (10–30 μM; Figure 5a).

Responses from TRPA1 could be modestly enhanced following brief pre-treatment with PP2 (Figure 5b) or Zn^2+^ plus 60 μM AITC (eliciting an average 1.5-fold increase in relative amplitude and reaching an average 24% of the ionophore-induced maximum). Responses from SH-SY5Y cells transfected with Y69C were very small. Likewise, responses from the experimental mutants Y97F, Y226F and YY654–655FF were also very small in SHSY5Y cells compared with HEK293 cells (Figure 5a) and could only be enhanced by pre-treatment with PP2 followed by high-dose AITC (60 μM; Figures 5a and 5b; Supplementary Figure S1b). In Figure 5(b), the effect of PP2 failed to reach statistical significance for TRPA1 N855S and R797T, similar to a lack of demonstrable effect on TRPA1 N855S when expressed in HEK293 cells (Figure 4). These particular variants are highly sensitive to TRPA1 agonist such that enhancement by PP2 pre-treatment may require more in-depth titration of both agonist and PP2 concentrations to achieve a combination of agents that leads to an enhanced iCa^2+^ signal amplitude [12]. (Untransfected SH-SY5Y cells did not exhibit intracellular Ca^2+^ signals in response to TRPA1 agonists, including AITC when added at concentrations up to 60 μM, in contrast with HEK293 cells; see Supplementary Data).

## DISCUSSION

In our survey of stably transfected HEK293 cell clones we found that non-conservative substitutions in the TRPM8 or TRPA1 N-terminal domain were generally associated with poor response to agonists. Only one of the variants studied, TRPA1 Y69C, exhibited good responses with high sensitivity to agonists. This variant was only marginally less sensitive to agonists than TRPA1 N855S and both appeared more sensitive than wild-type TRPA1 in measurements of apparent EC_50_. Interestingly, these two variants also appeared to be less sensitive to the antagonist A967079 than wild-type TRPA1. Determining whether these differences are due to structural changes in the protein or merely reflect an elevated level of channel protein expression will require further investigation, as discussed later.

Since all clones expressing the TRPM8 variants K423N and R475C responded relatively poorly to agonist treatment, one interpretation could be that each residue change may compromise formation of fully functional TRPM8 channels. However, such a conclusion must be considered alongside other possibilities. Poor levels of protein expression could simply be the cause of the reduced signal strengths (typified by relatively low iCa^2+^max observed in the intracellular Ca^2+^ assay). Therefore, further studies aimed at measuring levels of mutated channel protein and, of equal of importance, its sub-cellular localization [32] in transfected clones are required to interpret the current findings. However, this will most probably require use of epitope-tagged versions of each variant compared with tagged wild-type channel. Similar considerations apply to further studies of poorly responsive TRPA1 variants A366D and E477K cloned into HEK293 cells.

It will be interesting to investigate whether TRPA1 A366D, E477K or D573A are poorly expressed, retained in the endoplasmic reticulum or miss-localized elsewhere since these residues are thought to contribute to the formation of a putative regulatory EF hand structure [22] in the channel.

Responses to TRPM8 or TRPA1 agonists were modulated by pre-treatment of cells with micromolar PP2 or Zn^2+^ in a mutation- or clone-specific fashion. The specific findings are summarized in Table 1. Interestingly, PP2 inhibited responses from non-mutated TRPM8 and from TRPM8 R475C, but enhanced the responses from TRPM8 G150R, K423N and R485W. Thus PP2 may exert more than one effect on channels in cells. Perhaps it may bind directly to channels or it may indirectly cause channel modification by inhibiting phosphorylation at tyrosine residues. Alternatively, PP2 may alter vesicular trafficking such that more channel protein is delivered to areas accessible to gate Ca^2+^ ions.

Addition of Zn^2+^ had a similar effect to PP2 on the responses of TRPM8 variants to agonist, with the most noticeable enhancing effects on K423N and R485W. Rescue of TRPM8 mutants by Zn^2+^ is a novel observation, but with similarities to the effects of Zn^2+^ on mutated TRPA1 and suggestive of stabilization of miss-folded protein [10].

Weak responses to TRPA1 agonist from A366D or E477K could also be enhanced by pre-treating transfected HEK293 cells with PP2, similar to our previous observations in SH-SY5Y cells [12] or with Zn^2+^. Interestingly, micromolar concentrations of Zn^2+^ activate TRPA1 in patho-physiological settings [33,34].

When TRPA1 variant cDNA constructs were transfected into SH-SY5Y cells, the responses to TRPA1 agonists were smaller than those observed in transfected HEK293 cells, similar to our previous findings [12]. Interestingly, even N855S and Y69C appeared to be poorly expressed in SH-SY5Y relative to expression in HEK293. Also, individual tyrosine-to-phenylalanine mutations (Y97F, Y226F and YY654–655FF) did not improve responses of TRPA1 in SH-SY5Y cells. This is an interesting preliminary result in view of the potential for these tyrosine residues to be phosphorylation sites within TRPA1 (as suggested by NetPhos 2.0 and Scansite algorithms). A more in-depth screen would be required to identify sites of regulatory phosphorylation in TRPA1.

In summary, the present study indicates that further analyses of the properties of genetic variants affecting the structure of human TRPA1 and TRPM8 are required to identify candidates possessing altered activity. Altered channel activity may be an important pre-disposing property in particular disease states. Interestingly, a non-conservative substitution near the extreme N-terminal region of TRPA1 (Y69C), within AR1, was associated with good expression and increased sensitivity to agonist. Combined with the other findings, this suggests that genetic variants of TRPA1 and TRPM8 may be classified as ‘well expressed’, ‘poorly expressed’ or ‘salvageable’ in *in vitro* studies. The ability of PP2 and Zn^2+^ to enhance responses from particular variants with poor responses may be a useful tool. The *in vitro* classifications will be helpful in comparisons with other genetic variants of TRPA1 and TRPM8.

## Abbreviations

A967079: 4-fluorophenyl-2-methyl-1-pentene-3-one oxime
AITC: allyl-isothiocyanate
AMTB: *N*-3-aminopropyl-2-3-methylphenyl-methoxy-*N*-2-thienylmethylbenzamide
AR: ankyrin repeat
HEK: human embryonic kidney
RFU: relative fluorescence units
SNP: single nt polymorphism
WS 12: 4-methoxyphenyl-5-methyl-2-1-methylethyl-cyclohexanecaboxamide
